# Branched-chain amino acids promotes the repair of exercise-induced muscle damage *via* enhancing macrophage polarization

**DOI:** 10.3389/fphys.2022.1037090

**Published:** 2022-12-06

**Authors:** Yunfeng Dong, Xuejiao Zhang, Rui Miao, Wei Cao, Hao Wei, Wei Jiang, Ruirui Gao, Yanhui Yang, Haipeng Sun, Junqiang Qiu

**Affiliations:** ^1^ Department of Exercise Biochemistry, School of Sports Science, Beijing Sport University, Beijing, China; ^2^ Institute of Physical Education, Shanxi Datong University, Datong, China; ^3^ NHC Key Laboratory of Hormones and Development, Tianjin Key Laboratory of Metabolic Diseases, Chu Hsien-I Memorial Hospital & Tianjin Institute of Endocrinology, Tianjin Medical University, Tianjin, China; ^4^ Center for Cardiovascular Diseases, The Province and Ministry Co-Sponsored Collaborative Innovation Center for Medical Epigenetics, Tianjin Medical University, Tianjin, China; ^5^ Beijing Sports Nutrition Engineering Research Center, Beijing, China

**Keywords:** branched-chain amino acids, macrophage, exercise-induced muscle damage, mTORC1, muscle satellite cell

## Abstract

The repair of exercise-induced muscle damage (EIMD) is closely related with inflammation. Branched-chain amino acids (BCAAs), as a nutritional supplement, promote EIMD repair; however, the underlying mechanism remains unclear. *In vivo*, Sprague–Dawley rats were subjected to Armstrong’s eccentric exercise (a 120-min downhill run with a slope of −16° and a speed of 16 m min^−1^) to induce EIMD and BCAA supplement was administered by oral gavage. Protein expression of macrophages (CD68 and CD163) and myogenic regulatory factors (MYOD and MYOG) in gastrocnemius was analyzed. Inflammatory cytokines and creatine kinase (CK) levels in serum was also measured. *In vitro*, peritoneal macrophages from mice were incubated with lipopolysaccharide (LPS) or IL-4 with or without BCAAs in culture medium. For co-culture experiment, C2C12 cells were cultured with the conditioned medium from macrophages prestimulated with LPS or IL-4 in the presence or absence of BCAAs. The current study indicated BCAA supplementation enhanced the M1/M2 polarization of macrophages in skeletal muscle during EIMD repair, and BCAAs promoted M1 polarization through enhancing mTORC1-HIF1α-glycolysis pathway, and promoted M2 polarization independently of mTORC1. In addition, BCAA-promoted M1 macrophages further stimulated the proliferation of muscle satellite cells, whereas BCAA-promoted M2 macrophages stimulated their differentiation. Together, these results show macrophages mediate the BCAAs’ beneficial impacts on EIMD repair *via* stimulating the proliferation and differentiation of muscle satellite cells, shedding light on the critical role of inflammation in EIMD repair and the potential nutritional strategies to ameliorate muscle damage.

## Introduction

High intensity or unaccustomed strenuous exercise can cause exercise-induced muscle damage (EIMD). The main symptoms of EIMD are the loss of muscle function and delayed onset muscle soreness (Peake, 2019). The repair of EIMD mainly relies on muscle stem cells, termed satellite cells (SCs). After skeletal muscle damage, SCs proliferate and differentiate by expressing sequential transcription factors, such as Paired box7 (*Pax7*), Myogenic factor 5 (*Myf5*), Myoblast determination protein (*MYOD*), and Myogenin (*MYOG*) (Scala et al., 2021). Pax7 maintains the quiescent state of SCs. Myf5 and MYOD mainly regulate SCs proliferation, whereas MYOG controls SCs differentiation (Zammit, 2017). Understanding the mechanism underlying skeletal muscle repair and finding intervention strategies are important for accelerating the recovery process from EIMD.

Studies suggest the EIMD-induced inflammatory response is an integral part of the repair process (Fatouros and Jamurtas, 2016). Macrophages, with their inflammatory responses, play an important role in promoting skeletal muscle repair (Markus et al., 2021). Generally, macrophages divide into two types: The classically activated M1 macrophages and the alternatively activated M2 macrophages. M1 macrophages are present in the pro-inflammatory period of EIMD and associated with SCs proliferation (Otis et al., 2014; Varga et al., 2016), whereas M2 macrophages are present in the anti-inflammatory period of EIMD and associated with SCs differentiation and tissue repair process (Arnold et al., 2007; Fernandes et al., 2020). Importantly, M1 and M2 macrophages require different metabolic programs to support energy demands. M1 macrophages are mainly dependent on glycolysis metabolism, mediated by HIF1α (Wang et al., 2017), whereas M2 macrophages rely on fatty acid oxidation (FAO) and lipid metabolic reprogramming regulated by peroxisome proliferator-activated receptor γ (PPARγ) (Kang et al., 2018). This suggests that macrophage metabolism and polarization are closely linked, and macrophage polarization may be regulated by metabolic pathways.

Human studies have shown that Branched-Chain Amino Acids (BCAAs) supplementation is an effective approach to accelerate the recovery from EIMD (Rahimi et al., 2017; Owens et al., 2018). BCAAs, including leucine, isoleucine, and valine, are essential amino acids for mammals. BCAA, particularly leucine, activates the mammalian target of rapamycin complex 1 (mTORC1), a central signaling node that exerts widespread control over cellular metabolism and growth (Wolfson and Sabatini, 2017). Mammalian target of rapamycin (mTOR) plays an important role in the macrophages function by regulating gene expression at the transcriptional and translational levels (Kang and Kumanogoh, 2020). In recent years, BCAAs have been closely linked with glucose and lipid metabolism in metabolic and cardiovascular diseases (Wang et al., 2019; Supruniuk et al., 2021).

As a nutritional supplement, BCAAs reduce muscle soreness and the level of muscle damage biomarkers (Matsumoto et al., 2009; Doma et al., 2021; Khemtong et al., 2021; Bai et al., 2022), accompanied with changes in inflammatory cytokines in blood (Matsumoto et al., 2009). However, the underlying mechanism remains to be fully understood. In this study, we established an EIMD model and explored the role of macrophages in BCAA alleviated skeletal muscle damage. In addition, we further explored the mechanism of BCAA regulated macrophage polarization and the effect of BCAA-intervened macrophages on satellite cells.

## Materials and methods

### Animals

Male Sprague–Dawley rats (8-week-old; Charles River Laboratories China, Inc., Beijing, China) were provided with stanard rat chow and tap water *ad libitum* in a temperature-controlled room with a 12/12-h light/dark cycle. The protocol was approved by the Animal Research Ethics Committee of Beijing Sport University.

### High intensity exercise and BCAA administration

High intensity exercise refers to Armstrong’s eccentric exercise model published in 1983 (Armstrong et al., 1983) and has been reported to cause EIMD. All animals in exercise groups conducted adaptive training for 2 days (treadmill slope 0°, speed 16 m min^−1^, 5 min at day 1 and treadmill slope 0°, speed 16 m min^−1^, 10 min at day 2) and performed the formal exercise experiment after a day off. The Armstrong’s eccentric exercise was conducted on the fourth day (treadmill slope −16°, speed 16 m min^−1^, 120 min).

All animals in the BCAA group received BCAA supplement dissolved in water (1 g/kg body weight, consisted of 50% leucine, 25% isoleucine, and 25% valine) by oral gavage once a day from 3 days prior to the initiation of the eccentric exercise to the day they were sacrificed (Kato et al., 2016). On the day of eccentric exercise, BCAA supplement was administered immediately after the exercise. All animals in the placebo group (PLA) received distilled water by oral gavage once a day over the same period.

### Animal experiments

All animals were randomly divided into four groups: The non-injured control placebo (Con-PLA; n = 8), non-injured control BCAA (Con-BCAA; n = 8), exercise placebo (E-PLA; n = 32, and exercise BCAA supplement (E-BCAA; n = 32) groups. For the exercise group, 8 rats from each group were sacrificed at each time point of 1, 3, 5, and 7 days after the eccentric exercise. The gastrocnemius muscle and blood were collected at various time points. The gastrocnemius muscle was fixed using 4% paraformaldehyde for histochemical analysis, and blood was collected from abdominal aorta for enzyme-linked immunosorbent assay (ELISA). The remaining samples were stored at −80°C until protein expression analysis.

### ELISA to assess cytokine release in serum

The serum was collected from abdominal aorta of rats at each indicated time. Creatine kinase (CK), IL-6, and IL-10 levels in the diluted serum samples were analyzed using respective ELISA kits (Jianglaibio, shanghai, China) as per the manufacturer’s instructions. The final signals were read using a pan-wavelength micro plate reader (BioTek Instruments, United States).

### Muscle histology and immunohistochemistry

First, muscles samples were fixed in 4% paraformaldehyde and embedded in paraffin wax. Further, the samples were stained using hematoxylin and eosin (H&E). For immunohistochemical analysis, the sections were adhered to poly-L-lysine-coated slides. Further, the sections were deparaffinized and fixed in 0.1% trypsin for 30 min, followed by blocking with 5% serum for 30 min. The sections were incubated with the following primary antibodies for 4 h at room temperature: rabbit anti-mouse CD68 (1/300 dilution; CST) and rabbit anti-mouse CD163 (1/500 dilution; CST). Next, they were incubated with secondary antibodies [anti-rabbit FITC-conjugated (1/250 dilution, CST)] for 40 min at room temperature.

### Primary peritoneal macrophage culture and BCAA intervention

Primary peritoneal macrophages (M0) were isolated from mice as previously described (Bisgaard et al., 2016). 1ml of 3% Brewer thioglycollate medium was injected into the peritoneal cavity of each mouse. After 3 days, mouse was euthanized by cervical dislocation. 10 ml of cold PBS was injected into the peritoneal cavity of each mouse. The peritoneal fluid was collected and centrifuged for 10 min at 1,500 rpm in a refrigerated centrifuge. The cell pellet was resuspended in DMEM medium and cells were cultured for 4–6 h at 37°C, during which the peritoneal macrophages attached to culture plates, allowing their separation from other types of cells. Subsequently, non-adherent cells were removed by gently washing 3 times with warm PBS. The M0 macrophages were cultured in DMEM (Cell Science & Technology Institute, Japan) supplemented with 10% FBS (Gibco), 100 U/mL penicillin, and 100 μg/ml streptomycin. M1 and M2 macrophages were obtained by incubating with LPS (0.5 μg/ml, Sigma) and IL-4 (20 ng/ml, PeproTech), respectively, for 24 h. For BCAA intervention, 8 mmol/L BCAA stock solution (Sigma) was used. Macrophages were treated with different concentrations of BCAAs as previously described (Lian et al., 2015; Wang et al., 2019). Cells were treated with 2-deoxy-D-glucose (Liu et al., 2021) (2-DG, 1 mM; Solarbio, Shanghai, China) and rapamycin (Zhenyukh et al., 2017) (100 nM, Sigma-Aldrich).

### Co-culture of C2C12 cells and macrophages

M0 macrophages were stimulated with LPS or IL-4 in the presence or absence of BCAAs in culture medium for 24 h, respectively. The cells were then cultured with fresh medium for 12 h, and the culture medium were collected, namely, M1 conditioned medium, M1+BCAA conditioned medium, M2 conditioned medium, and M2+BCAA conditioned medium. For the proliferation assay, C2C12 cells were cultured in M1 conditioned medium and M1+BCAA conditioned medium, respectively, for 24 or 48 h. For the differentiation assay, differentiation medium (DMEM medium with 2% horse serum, 100 U/mL penicillin, and 100 μg/ml streptomycin) with M2 or M2+BCAA conditioned media (1:1) were added to the differentiating myotubes every 24 h for 1, 3, or 5 days. All cells were cultured in a humidified incubator at 37°C with 5% CO_2_ and 95% air.

### C2C12 cell proliferation assay

C2C12 cells proliferation was assessed by Cell Counting Kit-8 (CCK8, Dojindo, Japan). C2C12 cells were seeded in 96-well plate at density of 4 × 10^3^ cells/well and cultured with 100 μl M1 conditioned medium or 100 μl M1+BCAA conditioned medium for 48 h, respectively. 90 μl fresh DMEM medium and 10 μl CCK-8 reagent were then added to each well and the cells were incubated at 37°C for 1 h. The measurement was done using a pan-wavelength micro plate reader (BioTek Instruments, United States) at 450 nm.

### Real-time quantitative PCR

The total RNA were extracted from cells using Trizol reagent (Invitrogen, United States) and reverse transcribed into cDNA using RT SuperMix kit (Promega, United States). Real-time quantitative PCR (q-PCR) was performed using SYBR Green PCR mix (ABI, United States) and real-time PCR system (Bio-Rad) with the primer sequences in Table 1.

**TABLE 1:** Primers for quadriceps qPCR.

Gene	Forward primer	Reverse primer
18S	AGG​CCC​TGT​AAT​TGG​AAT​GAG​TC	GCT​CCC​AAG​ATC​CAA​CTA​CGA​G
IL-6	TAG​TCC​TTC​CTA​CCC​CAA​TTT​CC	TTG​GTC​CTT​AGC​CAC​TCC​TTC
TNF-α	CAG​GCG​GTG​CCT​ATG​TCT​C	CGA​TCA​CCC​CGA​AGT​TCA​GTA​G
iNOS	GCT​CGC​TTT​GCC​ACG​GAC​GA	AAG​GCA​GCG​GGC​ACA​TGC​AA
Arg-1	TGG​CTT​GCG​AGA​CGT​AGA​C	GCT​CAG​GTG​AAT​CGG​CCT​TTT
CD206	CTC​TGT​TCA​GCT​ATT​GGA​CGC	CGG​AAT​TTC​TGG​GAT​TCA​GCT​TC
Mgl1	AAC​CAA​TAG​CAG​CTG​CCT​TCA​TGC	TGC​AAC​AGC​TGA​GGA​AGG​ACT​TGA
Myf5	TAT​TAC​AGC​CTG​CCG​GGA​CA	CTG​CTG​TTC​TTT​CGG​GAC​CA
MYOG	GGT​GTG​TAA​GAG​GAA​GTC​TGT​G	TAG​GCG​CTC​AAT​GTA​CTG​GAT
Myh4	GAG​TTC​ATT​GAC​TTC​GGG​ATG​G	TGC​TGC​TCA​TAC​AGC​TTG​TTC​TTG
Hk2	CTG​CTT​TGG​AGA​TCC​GAG​GG	GTC​TAG​CTG​CTT​AGC​GTC​CC
PFK1	ACG​TGA​AGG​ATC​TGG​TGG​TTC	GGA​TTC​GGT​CGA​AGG​CTG​AA
LDHA	CGT​GCA​CTA​GCG​GTC​TCA​AA	GGA​GAT​CCA​TCA​TCT​CGC​CC

### Western blot analysis

The tissues or cells were harvested using lysis buffer and subjected to 10% SDS-PAGE. The separated proteins were transferred to polyvinylidene difluoride membranes and blocked using 5% bovine serum albumin for 1 h the membranes were incubated with the following primary antibodies for 12 h at 4°C: S6K (1/1000 dilution; CST), P-S6K-T389 (1/1000 dilution; CST), HIF1α (1/1000 dilution; Abcam), PPARγ (1/1000 dilution; CST), MYOD (1/1000 dilution; CST), MYOG (1/200 dilution; Abcam), and GAPDH (1/3000 dilution; CST). Further, they were incubated with the following secondary antibodies for 2 h at room temperature: HRP-labelled anti-mouse (1/5000, CST) or anti-rabbit (1/5000, Santa Cruz Biotechnology). Finally, the signal was detected using a Image Quant LAS 4000 system according to the manufacturer’s instructions.

### Statistical analysis

Statistical analyses were performed with one-way ANOVA or two-way ANOVA followed by Bonferroni’s multiple comparisons test using GraphPad Prism 9. All values are expressed as mean ± SEM. *p* < 0.05 was considered significant.

## Results

### BCAAs promote EIMD repair

**FIGURE 1:**
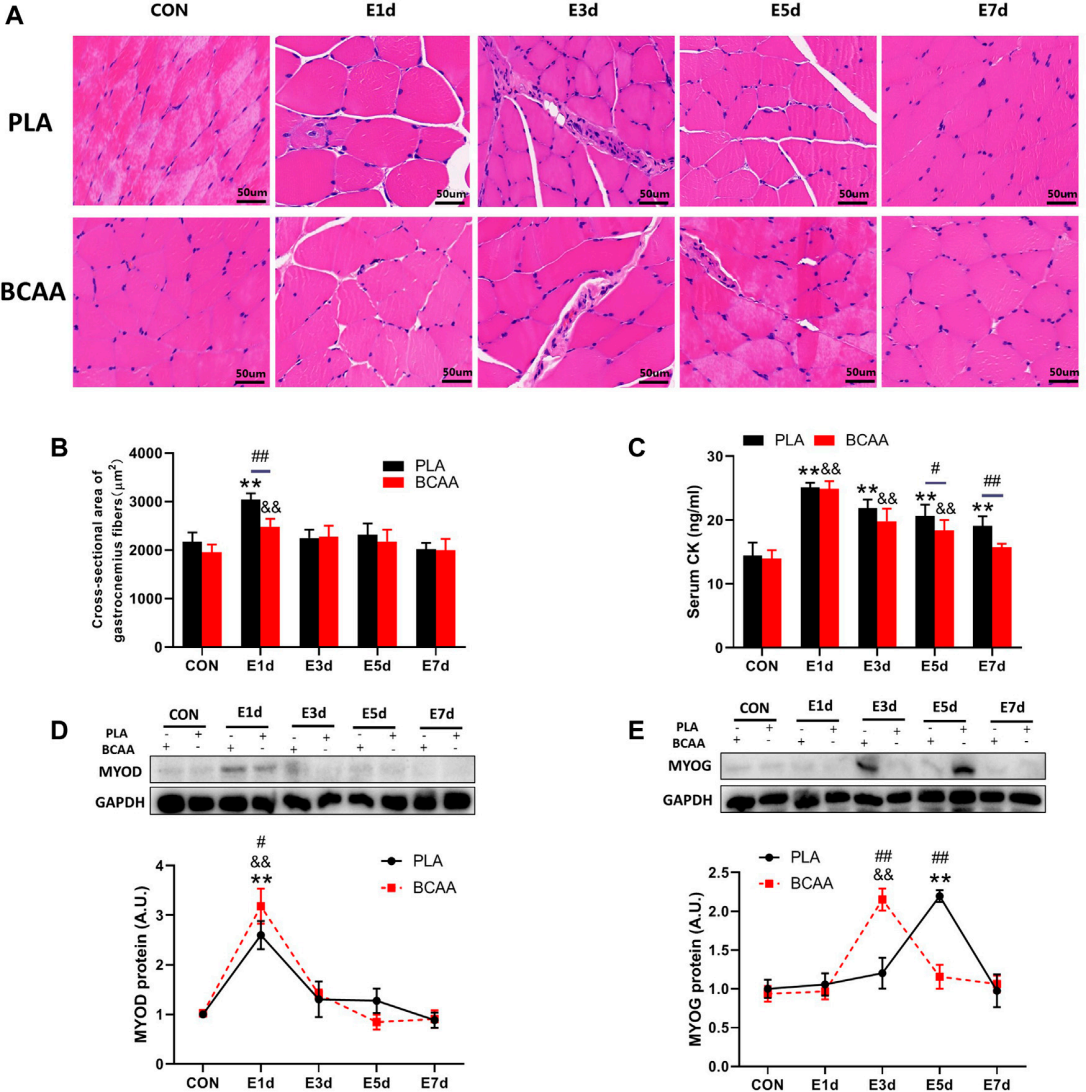
Branched-chain amino acids (BCAAs) promote the repair of exercise-induced muscle damage (EIMD). **(A)** The effect of BCAAs on the microstructure of gastrocnemius muscle in rats with EIMD as revealed by HE staining (Scale Bar = 50 um). **(B)** The effect of BCAAs on the cross-sectional area of gastrocnemius fibers in rats with EIMD. **(C)** The effect of BCAAs on serum CK levels in rats with EIMD. **(D,E)** The effect of BCAAs on the proliferation (MYOD) and differentiation (MYOG) of the gastrocnemius satellite cells (SCs) in rats with EIMD. Data are presented as mean ± SEM. ^*^ *p* < 0.05, ^**^ *p* < 0.05 vs. PLA CON; ^&^ *p* < 0.05, ^&&^ *p* < 0.05 vs. BCAA CON; ^#^ *p* < 0.05, ^##^ *p* < 0.05 between PLA and BCAA.

To explore the effect of BCAAs on EIMD, we established a rat model with EIMD, and treated the rats with or without BCAAs. The muscle fibers of the gastrocnemius in the PLA group showed varying degrees of swelling and dilated intercellular space after EIMD (Figures 1A,B). Moreover, the serum CK levels were increased after EIMD (Figure 1C). These changes indicated that the EIMD model was successfully constructed. Importantly, with BCAA treatment, the muscle fiber swelling was decreased on the first day after EIMD (Figure 1B) and the serum CK level was lower at the fifth and seventh day after EIMD (Figure 1C), compared with those in the PLA group. Meanwhile, the expression of SCs proliferation marker MYOD was increased in BCAA group (Figure 1D). In addition, BCAA advanced the peak expression of another SCs differentiation marker MYOG by 2 days, indicating that BCAA supplementation accelerated the repair of EIMD (Figure 1E). Collectively, these data demonstrated that BCAA supplementation promoted EIMD repair, consistent with previous studies (Kato et al., 2016; Doma et al., 2021).

### BCAAs promote M1/M2 polarization of macrophages during EIMD repair

**FIGURE 2:**
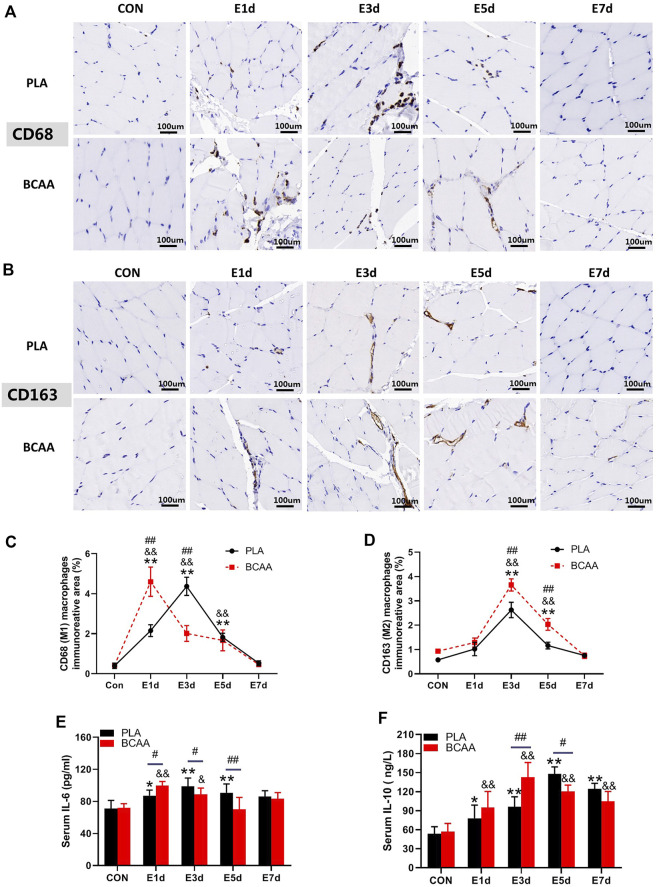
BCAAs promote M1 and M2 polarization of macrophages during EIMD repair. **(A–D)** BCAAs promoted CD68/CD163 expression in macrophages during EIMD repair as revealed by immunohistochemical analysis (Scale Bar = 100 um). **(E,F)** The effects of BCAAs on serum levels of inflammatory cytokines, namely, IL-6, and IL-10 during EIMD repair. Data are presented as mean ± SEM. ^*^ *p* < 0.05, ^**^ *p* < 0.01 vs. PLA CON; ^&^ *p* < 0.05, ^&&^ *p* < 0.01 vs. BCAA CON; ^#^ *p* < 0.05, ^##^ *p* < 0.01 between PLA and BCAA.

To explore the potential role of macrophages in the muscle repair facilitated by BCAAs, we analyzed the expression of marker proteins for M1/M2 polarization and the blood level of inflammatory cytokines in the EIMD animals. Immunohistochemical results revealed that BCAAs enhanced the protein expression of CD68 (M1) and CD163 (M2) during EIMD repair (Figures 2A–D). BCAA advanced the peak expression of CD68 by 2 days, suggesting that BCAAs accelerated macrophages M1 polarization during EIMD repair. Meanwhile, BCAAs increased the serum levels of proinflammatory cytokine IL-6 in the early stage of EIMD repair (Figure 2E) and the serum levels of anti-inflammatory cytokines IL-10 in the late stage (Figure 2F). Collectively, these data suggested that BCAAs promoted both M1 and M2 polarization of macrophages during EIMD repair.

### BCAA-promoted M1 macrophages enhance the proliferation of SCs

**FIGURE 3:**
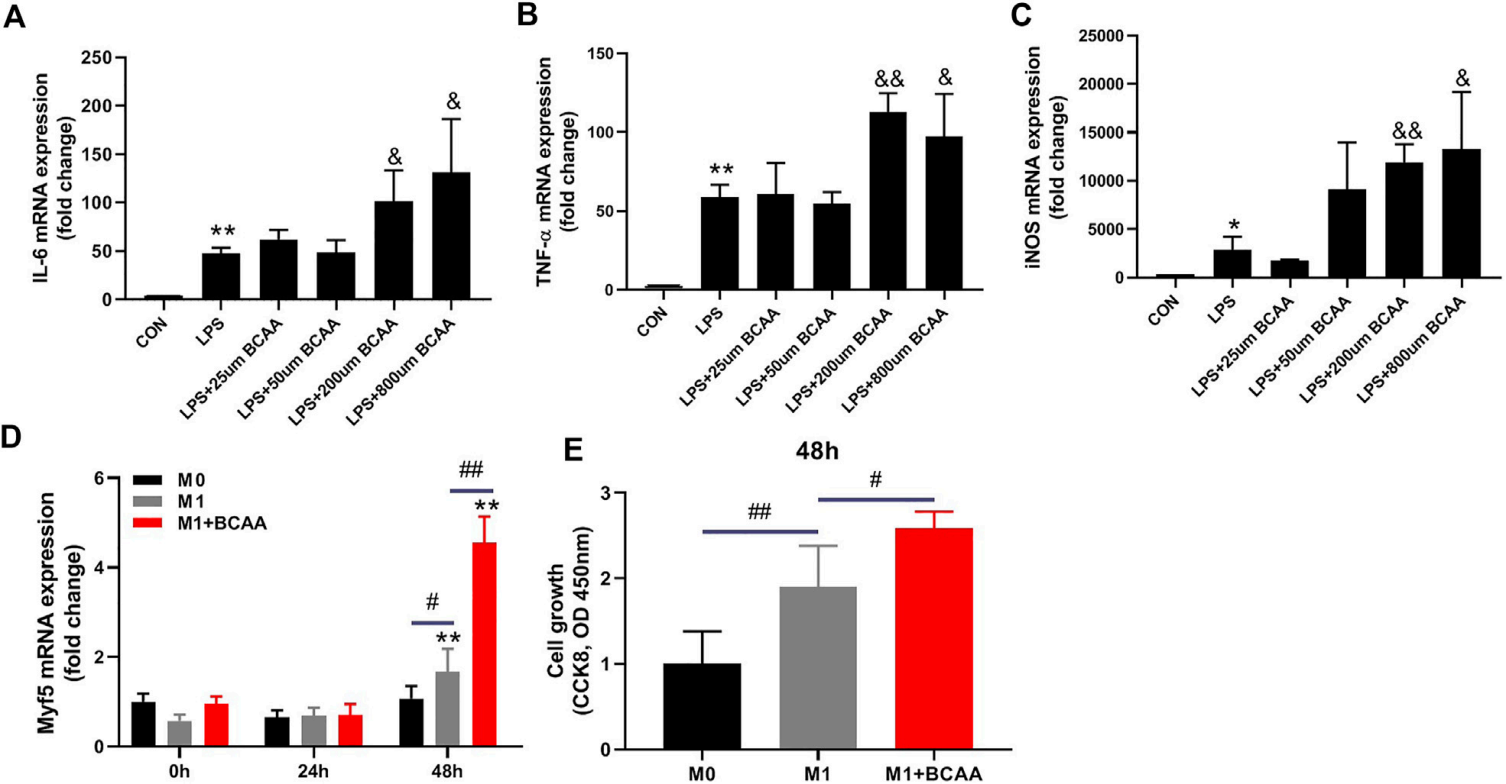
BCAA-promoted M1 macrophages enhance the proliferation of SCs. **(A–C)** The effects of various concentrations of BCAAs on the polarization of M1 macrophages. **(D,E)** The effect of BCAA (800 μm)-treated M1 macrophages on C2C12 proliferation. Data are presented as mean ± SEM. ^*^ *p* < 0.05, ^**^ *p* < 0.01 vs. CON/0h; ^&^ *p* < 0.05, ^&&^ *p* < 0.01 vs. LPS; ^#^ *p* < 0.05, ^##^ *p* < 0.01 between groups at the same time point.

We analyzed the direct effects of BCAAs on the polarization of M1 macrophages *in vitro*. We isolated the primary peritoneal macrophages from mice and exposed them to LPS with or without BCAAs in culture medium. 800μm BCAA promoted the mRNA expression of IL-6, TNF-α, and iNOS, suggesting BCAA enhanced M1 polarization (Figures 3A–C).

It has been suggested that M1 macrophage affects SCs proliferation (Minari and Thomatieli-Santos, 2022). We then tested whether the conditioned medium from M1 macrophages treated with BCAA could enhance SCs proliferation. To do so, M0 macrophages were prestimulated with LPS with or without BCAA for 24 h. Fresh medium was then added to the M1 macrophages for 12 h and collected as conditioned media. C2C12 cells were then cultured with these conditioned media for 24 or 48 h. As expected, medium from M1 macrophages promoted the mRNA expression of Myf5, which is further enhanced by BCAA-promoted M1 macrophages (Figure 3D). Further, we analyzed the proliferation of C2C12 cells cultured with conditioned medium using the CCK8 kit. The result showed that the conditioned medium from prestimulated M1 macrophages promoted C2C12 cells growth, which was further enhanced by the conditioned medium from BCAA-treated M1 macrophages (Figure 3E). Collectively, these data suggested that BCAA-promoted M1 macrophages could enhance the proliferation of SCs.

### BCAA-promoted M2 macrophages enhance the differentiation of SCs

**FIGURE 4:**
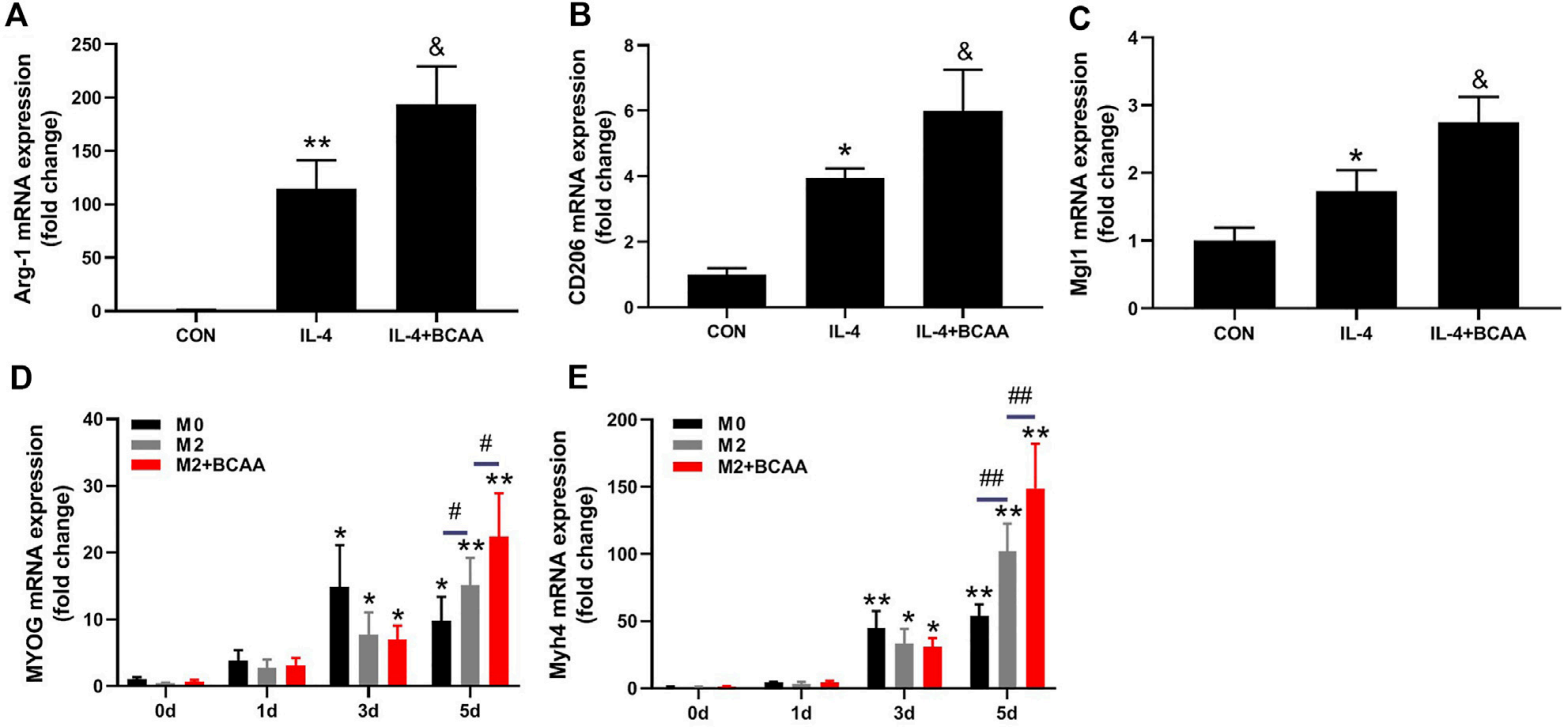
BCAA-promoted M2 macrophages enhance the differentiation of SCs. **(A–C)** The effect of BCAAs (800 μm) on M2 polarization of macrophages. **(D,E)** The effect of BCAA (800 μm)-treated M2 macrophages on C2C12 differentiation. Data are presented as mean ± SEM. ^*^ *p* < 0.05, ^**^ *p* < 0.01 vs. CON/0d; ^&^ *p* < 0.05, ^&&^ *p* < 0.01 vs. IL-4; ^#^ *p* < 0.05, ^##^ *p* < 0.01 between groups at the same time point.

We also explored the direct effects of BCAAs on the polarization of M2 macrophages *in vitro*. The primary peritoneal macrophages were isolated from mice and exposed to IL-4 with or without BCAAs in culture medium. 800 μm BCAA promoted the mRNA expression of Arg-1, CD206 and Mgl1, suggesting BCAA promoted M2 polarization (Figures 4A–C).

It has been suggested that M2 affects SC differentiation (Minari and Thomatieli-Santos, 2022). We then tested whether the conditioned medium from BCAA-promoted M2 macrophages could affect SCs differentiation. C2C12 cells were cultured with differentiation medium and conditioned media (1:1) from M2 or BCAA-treated M2 for 24 or 48 h. As expected, the conditioned medium from M2 macrophages promoted the mRNA expression of MYOG and myosin heavy chain 4 (Myh4) in C2C12 cells, which was further enhanced by the conditioned medium from BCAA-promoted M2 macrophages (Figures 4D,E). Collectively, these data indicated that BCAA-promoted M2 macrophages enhanced the differentiation of SCs.

### BCAAs promote M1 polarization *via* mTORC1-HIF1α-glycolysis pathway

**FIGURE 5:**
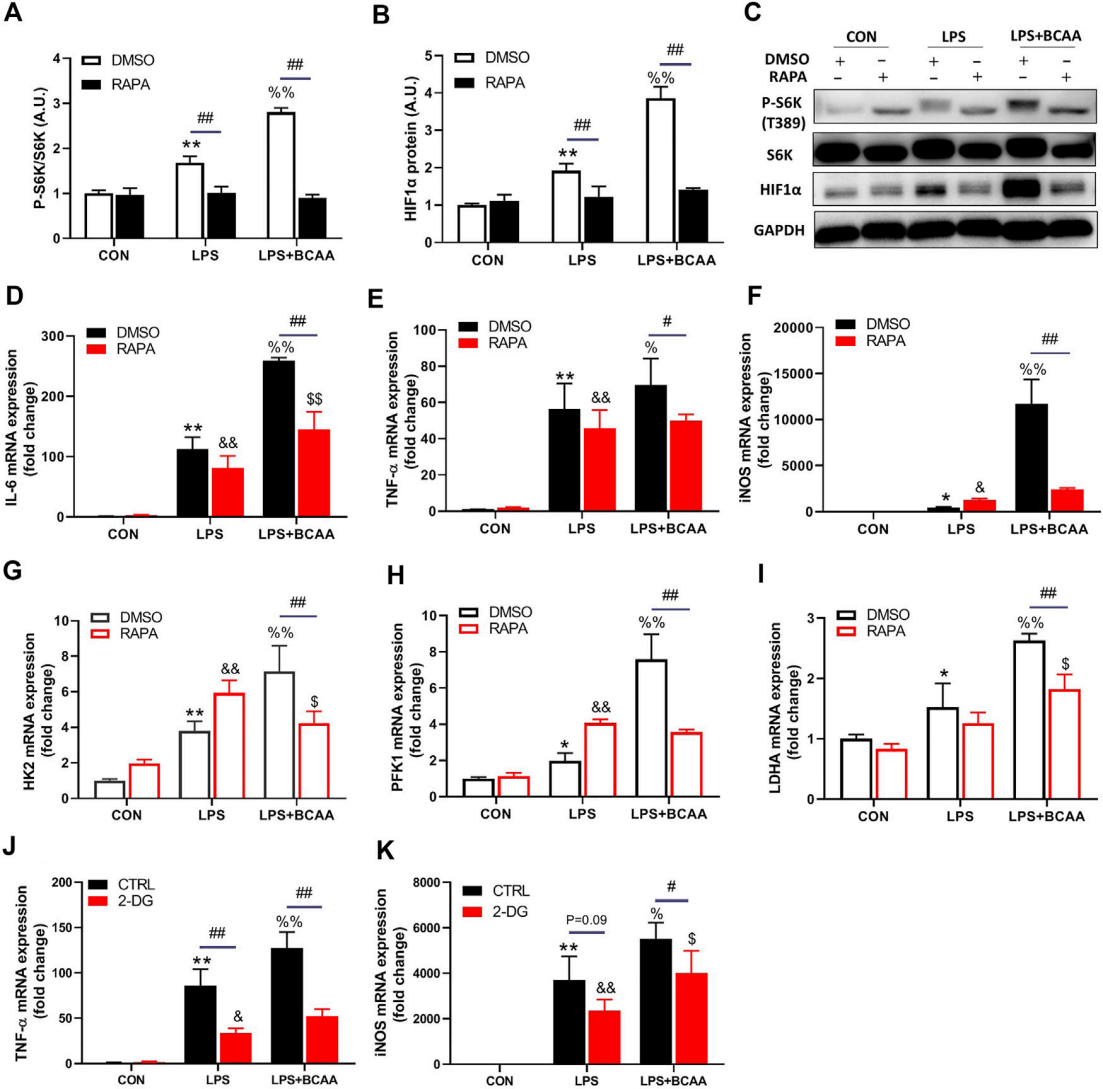
BCAAs promote M1 polarization *via* mTORC1-HIF1α-glycolysis pathway. **(A–C)** BCAAs (800 μm) promoted the expression of HIF1α and mTORC1 activation during M1 polarization, and the effect of RAPA (rapamycin, mTORC1 inhibitor) on the expression of HIF1α. **(D–F)** Changes of M1 polarization with RAPA. **(G–I)** The effect of RAPA on the rate-limiting enzyme of glycolysis. **(J,K)** Changes of M1 polarization with 2-DG, a glycolysis inhibitor. Data are presented as mean ± SEM. ^*^ *p* < 0.05, ^**^ *p* < 0.01 vs. CON/DMSO CON/CTRL CON; ^&^ *p* < 0.05, ^&&^ *p* < 0.01 vs. RAPA/2-DG Con; ^%^ *p* < 0.05, ^%%^ *p* < 0.01 vs. LPS/DMSO LPS/CTRL LPS; ^$^ *p* < 0.05, ^$$^ *p* < 0.01 vs. RAPA/2-DG LPS; ^#^ *p* < 0.05, ^##^ *p* < 0.01 between groups LPS or LPS + BCAA.

Next, we investigated how BCAA promoted M1 polarization. M1 macrophages are essentially glycolytic cells (Juban and Chazaud, 2017). mTORC1 is a central regulator of cellular metabolism, including glycolysis, and can be activated by BCAA. We then investigated the role of mTORC1 in the BCAA-promoted M1 polarization. As expected, BCAAs enhanced the activity of mTORC1 in the process of M1 polarization (Figures 5A–C). Importantly, rapamycin, an mTORC1 inhibitor, abolished BCAA-promoted M1 polarization (Figures 5D–F), suggesting mTORC1 mediated BCAA’s effect.

Hypoxia-inducible factor 1α (HIF1α), a transcriptional factor targeted by mTORC1, controls glycolysis and M1 polarization (Wang et al., 2017). BCAAs increased the protein level of HIF1α during M1 polarization (Figures 5B,C). Rapamycin abolished the BCAA-induced HIF1α and glycolytic enzymes expression (HK2, PFK1 and LDHA) (Figures 5B,G–I). Furthermore, 2-DG, the inhibitor of glycolysis, attenuated the mRNA levels of TNF-α and iNOS induced by BCAA (Figures 5J,K), suggesting a weakened M1 polarization. Collectively, these data demonstrated that BCAAs promoted M1 polarization *via* activating the mTORC1-HIF1α-glycolysis pathway.

### BCAAs promote M2 polarization independent of the mTORC1-PPARγ pathway

**FIGURE 6:**
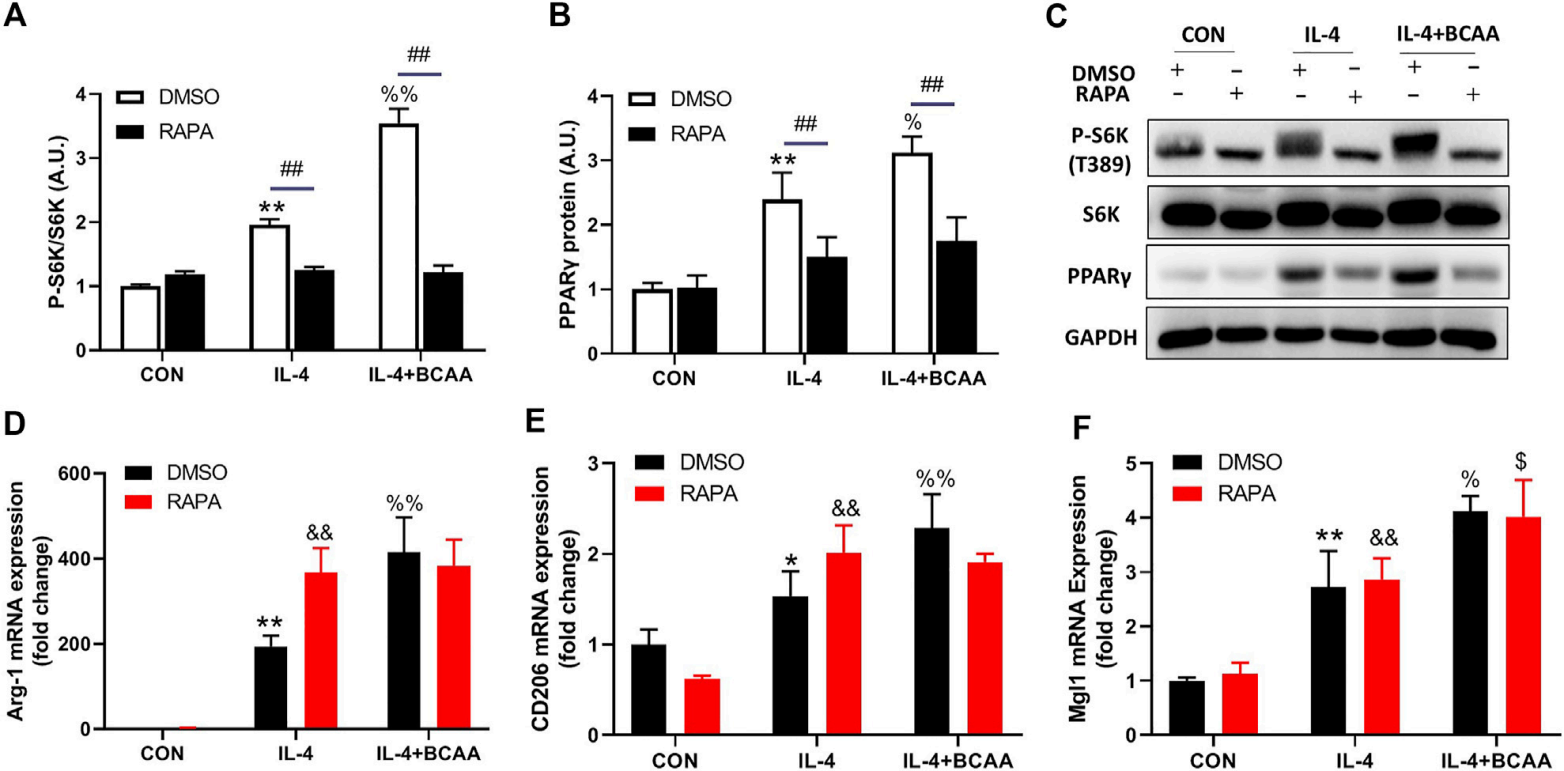
BCAAs promote M2 polarization independent of the mTORC1-PPARγ pathway. **(A–C)** BCAAs (800 μm) enhanced the expression of PPARγ and mTORC1 activity during M2 polarization, and the effect of RAPA (rapamycin, mTORC1 inhibitor) on the expression of PPARγ. **(D–F)** The effect of RAPA on the change in M2 polarization. Data are expressed as mean ± SEM. ^*^ *p* < 0.05, ^**^ *p* < 0.01 vs. CON/DMSO CON; ^&^ *p* < 0.05, ^&&^ *p* < 0.01 vs. RAPA CON; ^%^ *p* < 0.05, ^%%^ *p* < 0.01 vs. IL-4/DMSO IL-4; ^$^ *p* < 0.05, ^$$^ *p* < 0.01 vs. RAPA IL-4; ^#^ *p* < 0.05, ^##^ *p* < 0.01 between groups IL-4 or IL-4+BCAA.

We also explored how BCAA promoted M2 polarization. Lipid metabolic reprogramming is essential for M2 polarization (van den Bosch et al., 2017). mTORC1 and its downstream transcription factor PPARγ are key regulators of lipid metabolism in M2 polarization. We then investigated the role of mTORC1 and PPARγ in the BCAA-promoted M2 polarization. As expected, BCAAs enhanced the activity of mTORC1 and the protein level of PPARγ during M2 polarization (Figures 6A,B). Interestingly, rapamycin abolished the BCAA-induced mTORC1 activation and PPARγ expression (Figure 6C), but showed no effect on BCAA-promoted M2 polarization (Figures 6D–F). Collectively, these data suggested that BCAAs promoted M2 polarization independent of the mTORC1-PPARγ pathway.

## Discussion

In the present study, we demonstrated BCAA supplementation promotes the repair of EIMD *via* enhancing macrophage polarization. M1 and M2 macrophages stimulate the proliferation and differentiation of muscle satellite cells, respectively. mTORC1-HIF1α-glycolysis pathway mediates the BCAA’s effect on M1 polarization while BCAA-promoted M2 polarization is independent of mTORC1.

Previous studies have shown that BCAA supplementation alleviate the level of CK and muscle soreness following EIMD in human. In the present study, our results also indicated that BCAA supplementation reduce the level of CK and accelerate the recovery of damaged muscle fibers, which is consistent with previous studies (Fouré and Bendahan, 2017; Doma et al., 2021), however, the mechanism remains unclear. Kato et al. (2016) study further suggested that leucine-enriched essential amino acids reduce muscle inflammation and enhance muscle repair after eccentric contraction in rats. This phenomenon suggested that BCAA-improved EIMD may be related to inflammation. Previous studies indicated that macrophages play an important role during skeletal muscle repair (Juhas et al., 2018; De Santa et al., 2019; Fernandes et al., 2020). There is no relevant study on whether BCAA intervention affect macrophage polarization during EIMD. Our results showed that BCAA enhances M1 and M2 polarization during skeletal muscle repair in different time stages, meanwhile, similar changes in the serum levels of inflammatory factors were observed *in vitro*. These data suggested that macrophage polarization plays an important role in BCAA-induced muscle repair.

In addition, skeletal muscle repair is a complex biological process, the activation, proliferation, and differentiation of SCs provides the potential capacity to muscle repair (La et al., 2018). In the current study, our results showed that BCAA supplementation promotes the proliferation and differentiation of skeletal muscle SCs, which is consistent with previous study (Duan et al., 2017). However, Kato et al. (2016) have reported that BCAAs does not affect SCs. This discrepancy could be attributed to the different muscle damage models. Muscle damage is induced by high-intensity eccentric exercise in the current study while electric stimulation of the tibialis anterior muscle is used to induce EIMD in anesthetized rats in Kato’s study. Further experiments are warranted to analyze the differences between these two muscle damage models.

Our results show BCAAs promote M1 and M2 polarization of macrophages, which further promote the proliferation and differentiation of SCs, respectively. Previous studies have reported that the pro-inflammatory and anti-inflammatory factors promote the proliferation and differentiation of SCs, respectively (Akahori et al., 2015; Varga et al., 2016). It can be speculated that BCAA-promoted pro- and anti-inflammatory factors mediate the stimulation of SC proliferation and differentiation from M1 and M2 macrophages. Meanwhile, other possible mechanisms should also be considered. The repair process of skeletal muscle involves a variety of cell types, such as macrophages and SCs (Tidball, 2017). It is not entirely clear how macrophages and SCs work together to promote skeletal muscle repair. Shang et al. (2020) have reported that macrophage-derived glutamine promotes satellite cell and muscle regeneration. Due to the important relationship between BCAA and glutamine, we speculate that glutamine may play an important role in BCAA-promoted macrophages stimulating SCs and muscle repair.

mTOR signaling pathway is an important link between immune response and cell metabolism, moreover, mTORC1 is an important factor in sensing intracellular amino acid concentration (Kang and Kumanogoh, 2020). In the present study, our results also show that BCAAs promote M1 polarization dependent on mTORC1-HIF1α-glycolysis pathway, whereas BCAAs promote M2 polarization independent of the mTORC1/PPARγ pathway. Previous studies show that mTORC1 plays an inconsistent role in M2 polarization. One study indicates that IL-4 stimulated bone marrow-derived macrophages (BMDM) from TSC1 knockout mice, which increased mTORC1 activity, M2 polarization weakened (Byles et al., 2013). However, two other studies showed IL-4 stimulated BMDM with mTOR inhibition (Torin1) or Raptor knockout mice (decreased mTORC1 activity), M2 polarization weakened (Covarrubias et al., 2016; Kimura et al., 2016). The reasons may be related to different the knockout mice model. In addition, Covarrubias et al. (2016) have reported that BMDMs were treated with IL-4 for 16 h with 2-DG (the glycolysis inhibitor) or etomoxir (the β-oxidation inhibitor), the mRNA levels of M2 polarization decreased. This phenomenon suggests the metabolism in M2 polarization is not only lipid metabolism and may also involve glycolysis. Therefore, further experiments are warranted to explore the mechanism how BCAA regulates M2 polarization.

It has been shown that there is a wide spectrum of macrophage activation states *in vivo* (Murray et al., 2014; Dort et al., 2019). An expanded range of stimuli can drive macrophage activation with distinct activation profiles in different directions (Kang and Kumanogoh, 2020). *In vitro*, M1/M2 polarization can be stimulated by chemical drugs or specific stimuli. Of note, the M1/M2 polarization *in vitro* ignores the complexity of stimuli and microenvironment and the wide activation spectrum *in vivo*. In the current study, we used chemically activated macrophages to investigate the effect of BCAA on macrophage polarization *in vitro*. The results of cultured cells were consistent with the expression of macrophage activation markers *in vivo*. Whether the macrophage polarization *in vitro* accurately recapitulates the changes of macrophages *in vivo* remains to be fully determined. Staining of different types of macrophages in the damaged muscle may be of help.

The current study demonstrated that BCAAs promoted C2C12 proliferation and differentiation *via* enhancing the macrophage polarization *in vitro*. On the other hand, when BCAAs intake was increased *in vivo*, the SCs were exposed to a large number of factors, including the elevated BCAAs and inflammatory factors. Whether BCAAs exert direct impacts on myoblasts remains to be investigated. It is also possible that there are additive impacts from elevated BCAAs and inflammatory factors. Nevertheless, our data show that macrophages mediate, at least partially, the impacts of BCAAs on SCs and thus the repair of EIMD. One limitation of our study is that, although we demonstrate that BCAAs promote the function of SCs *via* macrophages in cultured cells, direct evidence for this occurring *in vivo* is lacking, which could be provided by macrophage depletion animal model and the staining for SCs to assess their numbers in the damaged muscle.

In summary, the current study shows that BCAAs improve EIMD repair by promoting the proliferation and differentiation of muscle SCs through macrophage polarization. The results highlight the critical role of macrophage in BCAA-induced repair of EIMD and indicate new approaches for the treatment of muscle-related diseases.

## Data Availability

The raw data supporting the conclusion of this article will be made available by the authors, without undue reservation.
